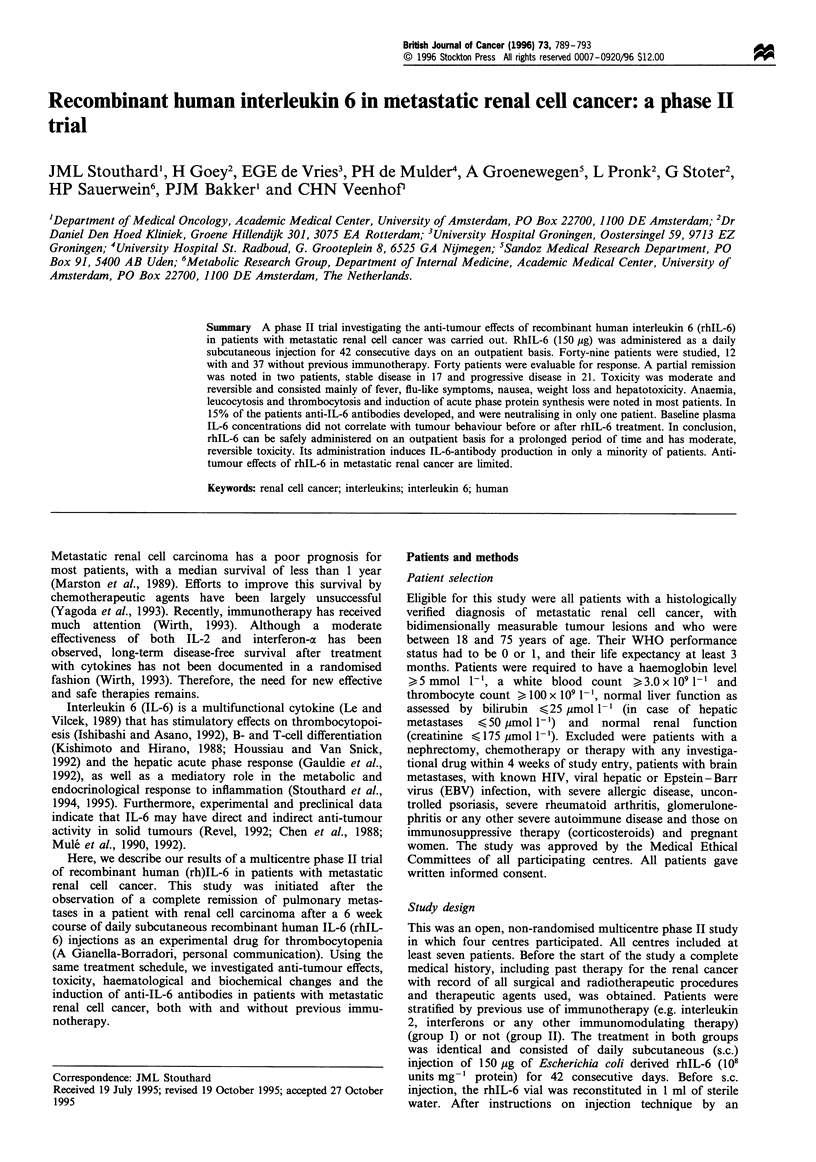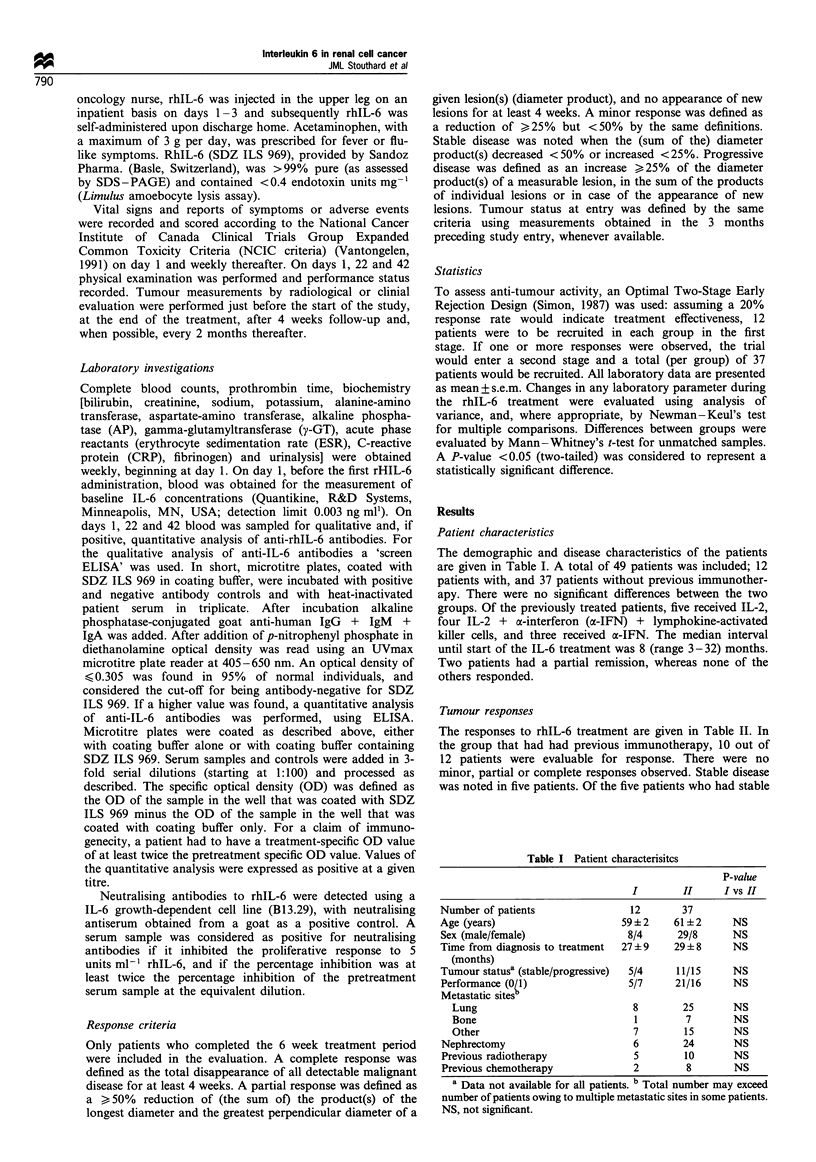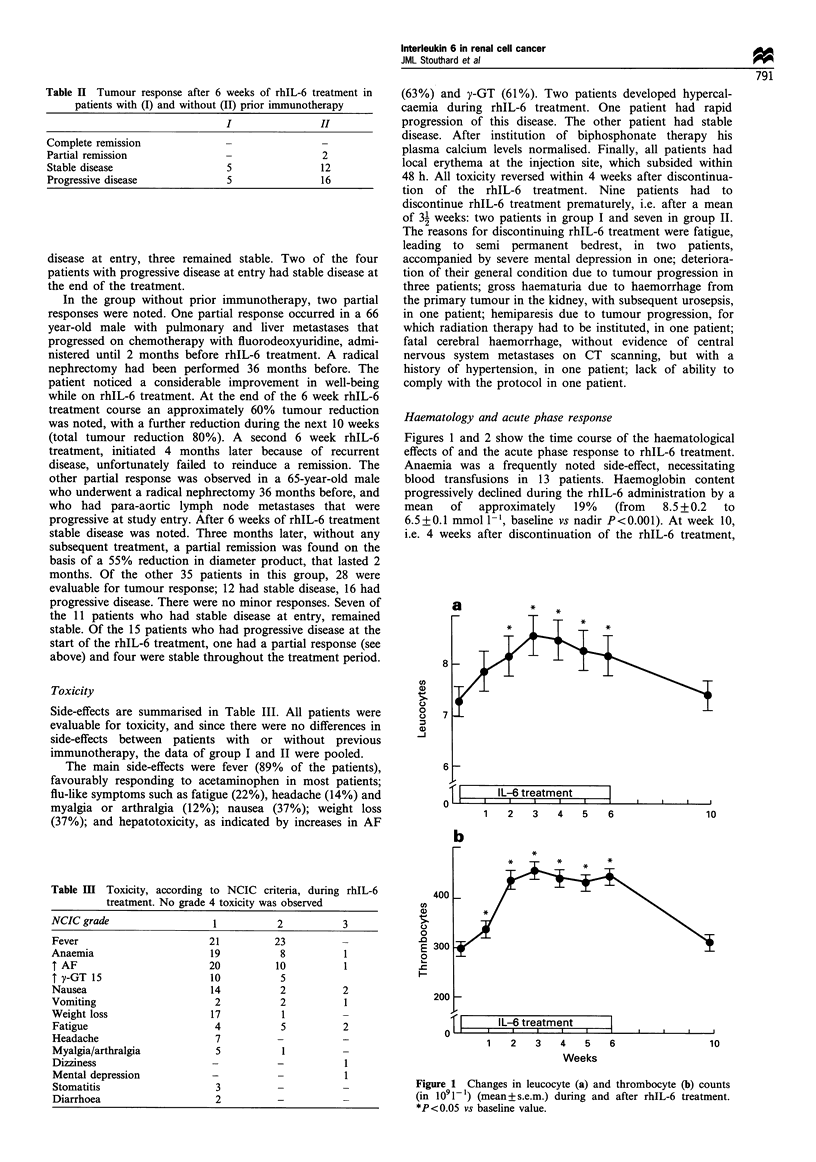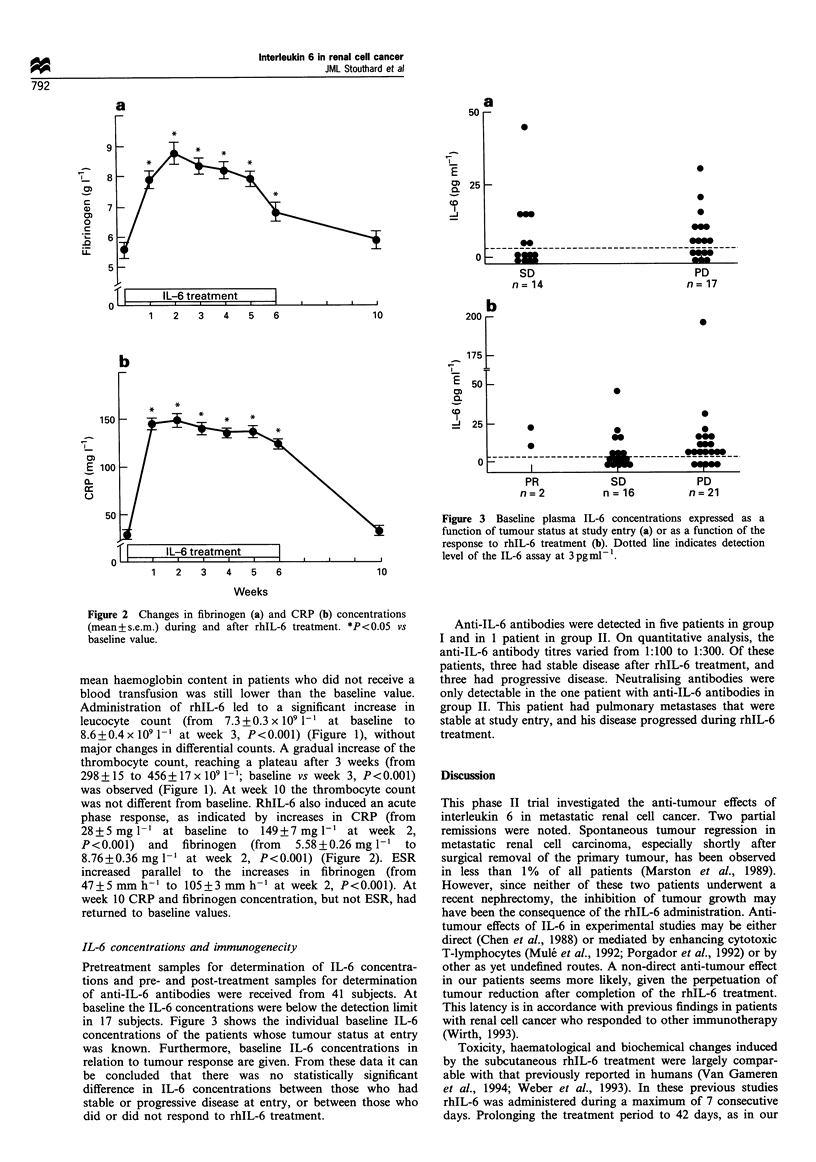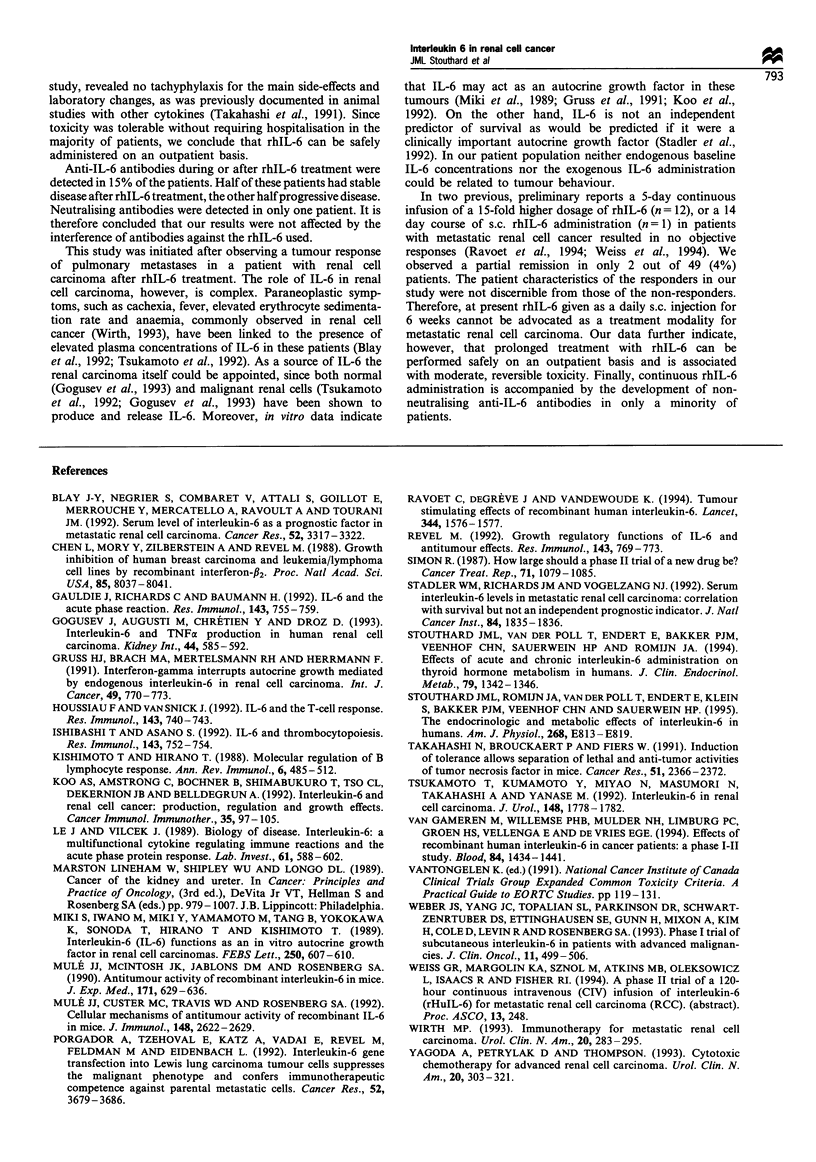# Recombinant human interleukin 6 in metastatic renal cell cancer: a phase II trial.

**DOI:** 10.1038/bjc.1996.137

**Published:** 1996-03

**Authors:** J. M. Stouthard, H. Goey, E. G. de Vries, P. H. de Mulder, A. Groenewegen, L. Pronk, G. Stoter, H. P. Sauerwein, P. J. Bakker, C. H. Veenhof

**Affiliations:** Department of Medical Oncology, Academic Medical Center, University of Amsterdam, The Netherlands.

## Abstract

A phase II trial investigating the anti-tumour effects of recombinant human interleukin 6 (rhIL-6) in patients with metastatic renal cell cancer was carried out. RhIL-6 (150 microgram) was administered as a daily subcutaneous injection for 42 consecutive days on an outpatient basis. Forty-nine patients were studied, 12 with and 37 without previous immunotherapy. Forty patients were evaluable for response. A partial remission was noted in two patients, stable disease in 17 and progressive disease in 21. Toxicity was moderate and reversible and consisted mainly of fever, flu-like symptoms, nausea, weight loss and hepatotoxicity. Anaemia, leucocytosis and thrombocytosis and induction of acute phase protein synthesis were noted in most patients. In 15% of the patients anti-IL-6 antibodies developed, and were neutralising in only one patient. Baseline plasma IL-6 concentrations did not correlate with tumour behaviour before or after rhIL-6 treatment. In conclusion, rhIL-6 can be safely administered on an outpatient basis for prolonged period of time and has moderate, reversible toxicity. Its administration induces IL-6-antibody production in only a minority of patients. Antitmour effects of rhIL-6 in metastatic renal cancer are limited.


British Journal of Cancer (1996) 73, 789-793
© 1996 Stockton Press All rights reserved 0007-0920/96 $12.00

### Recombinant human interleukin 6 in metastatic renal cell cancer: a phase II trial

JML Stouthard[1], H Goey[2], EGE de Vries[3], PH de Mulder[4], A Groenewegen[5], L Pronk[2], G Stoter[2], HP Sauerwein[6], PJM Bakker[1] and CHN Veenhof[1]

[1]*Department of Medical Oncology, Academic Medical Center, University of Amsterdam, PO Box 22700, 1100 DE Amsterdam;* [2]*Dr Daniel Den Hoed Kliniek, Groene Hillendijk 301, 3075 EA Rotterdam;* [3]*University Hospital Groningen, Oostersingel 59, 9713 EZ Groningen;* [4]*University Hospital St. Radboud, G. Grooteplein 8, 6525 GA Nijmegen;* [5]*Sandoz Medical Research Department, PO Box 91, 5400 AB Uden;* [6]*Metabolic Research Group, Department of Internal Medicine, Academic Medical Center, University of Amsterdam, PO Box 22700, 1100 DE Amsterdam, The Netherlands.*

**Summary** A phase II trial investigating the anti-tumour effects of recombinant human interleukin 6 (rhIL-6) in patients with metastatic renal cell cancer was carried out. RhIL-6 (150 $\mu$g) was administered as a daily subcutaneous injection for 42 consecutive days on an outpatient basis. Forty-nine patients were studied, 12 with and 37 without previous immunotherapy. Forty patients were evaluable for response. A partial remission was noted in two patients, stable disease in 17 and progressive disease in 21. Toxicity was moderate and reversible and consisted mainly of fever, flu-like symptoms, nausea, weight loss and hepatotoxicity. Anaemia, leucocytosis and thrombocytosis and induction of acute phase protein synthesis were noted in most patients. In 15% of the patients anti-IL-6 antibodies developed, and were neutralising in only one patient. Baseline plasma IL-6 concentrations did not correlate with tumour behaviour before or after rhIL-6 treatment. In conclusion, rhIL-6 can be safely administered on an outpatient basis for a prolonged period of time and has moderate, reversible toxicity. Its administration induces IL-6-antibody production in only a minority of patients. Anti-tumour effects of rhIL-6 in metastatic renal cancer are limited.

**Keywords:** renal cell cancer; interleukins; interleukin 6; human

Metastatic renal cell carcinoma has a poor prognosis for most patients, with a median survival of less than 1 year (Marston *et al.*, 1989). Efforts to improve this survival by chemotherapeutic agents have been largely unsuccessful (Yagoda *et al.*, 1993). Recently, immunotherapy has received much attention (Wirth, 1993). Although a moderate effectiveness of both IL-2 and interferon-$\alpha$ has been observed, long-term disease-free survival after treatment with cytokines has not been documented in a randomised fashion (Wirth, 1993). Therefore, the need for new effective and safe therapies remains.

Interleukin 6 (IL-6) is a multifunctional cytokine (Le and Vilcek, 1989) that has stimulatory effects on thrombocytopoiesis (Ishibashi and Asano, 1992), B- and T-cell differentiation (Kishimoto and Hirano, 1988; Houssiau and Van Snick, 1992) and the hepatic acute phase response (Gauldie *et al.*, 1992), as well as a mediatory role in the metabolic and endocrinological response to inflammation (Stouthard *et al.*, 1994, 1995). Furthermore, experimental and preclinical data indicate that IL-6 may have direct and indirect anti-tumour activity in solid tumours (Revel, 1992; Chen *et al.*, 1988; Mulé *et al.*, 1990, 1992).

Here, we describe our results of a multicentre phase II trial of recombinant human (rh)IL-6 in patients with metastatic renal cell cancer. This study was initiated after the observation of a complete remission of pulmonary metastases in a patient with renal cell carcinoma after a 6 week course of daily subcutaneous recombinant human IL-6 (rhIL-6) injections as an experimental drug for thrombocytopenia (A Gianella-Borradori, personal communication). Using the same treatment schedule, we investigated anti-tumour effects, toxicity, haematological and biochemical changes and the induction of anti-IL-6 antibodies in patients with metastatic renal cell cancer, both with and without previous immunotherapy.

Correspondence: JML Stouthard
Received 19 July 1995; revised 19 October 1995; accepted 27 October 1995

### Patients and methods

### *Patient selection*

Eligible for this study were all patients with a histologically verified diagnosis of metastatic renal cell cancer, with bidimensionally measurable tumour lesions and who were between 18 and 75 years of age. Their WHO performance status had to be 0 or 1, and their life expectancy at least 3 months. Patients were required to have a haemoglobin level $\geqslant 5$ mmol $l^{-1}$, a white blood count $\geqslant 3.0 \times 10^9$ $l^{-1}$ and thrombocyte count $\geqslant 100 \times 10^9$ $l^{-1}$, normal liver function as assessed by bilirubin $\leqslant 25$ $\mu$mol $l^{-1}$ (in case of hepatic metastases $\leqslant 50$ $\mu$mol $l^{-1}$) and normal renal function (creatinine $\leqslant 175$ $\mu$mol $l^{-1}$). Excluded were patients with a nephrectomy, chemotherapy or therapy with any investigational drug within 4 weeks of study entry, patients with brain metastases, with known HIV, viral hepatic or Epstein-Barr virus (EBV) infection, with severe allergic disease, uncontrolled psoriasis, severe rheumatoid arthritis, glomerulonephritis or any other severe autoimmune disease and those on immunosuppressive therapy (corticosteroids) and pregnant women. The study was approved by the Medical Ethical Committees of all participating centres. All patients gave written informed consent.

### *Study design*

This was an open, non-randomised multicentre phase II study in which four centres participated. All centres included at least seven patients. Before the start of the study a complete medical history, including past therapy for the renal cancer with record of all surgical and radiotherapeutic procedures and therapeutic agents used, was obtained. Patients were stratified by previous use of immunotherapy (e.g. interleukin 2, interferons or any other immunomodulating therapy) (group I) or not (group II). The treatment in both groups was identical and consisted of daily subcutaneous (s.c.) injection of 150 $\mu$g of *Escherichia coli* derived rhIL-6 ($10^8$ units $mg^{-1}$ protein) for 42 consecutive days. Before s.c. injection, the rhIL-6 vial was reconstituted in 1 ml of sterile water. After instructions on injection technique by an oncology nurse, rhIL-6 was injected in the upper leg on an inpatient basis on days 1–3 and subsequently rhIL-6 was self-administered upon discharge home. Acetaminophen, with a maximum of 3 g per day, was prescribed for fever or flu-like symptoms. RhIL-6 (SDZ ILS 969), provided by Sandoz Pharma. (Basle, Switzerland), was >99% pure (as assessed by SDS–PAGE) and contained <0.4 endotoxin units $mg^{-1}$ (*Limulus* amoebocyte lysis assay).

Vital signs and reports of symptoms or adverse events were recorded and scored according to the National Cancer Institute of Canada Clinical Trials Group Expanded Common Toxicity Criteria (NCIC criteria) (Vantongelen, 1991) on day 1 and weekly thereafter. On days 1, 22 and 42 physical examination was performed and performance status recorded. Tumour measurements by radiological or clinial evaluation were performed just before the start of the study, at the end of the treatment, after 4 weeks follow-up and, when possible, every 2 months thereafter.

*Laboratory investigations*

Complete blood counts, prothrombin time, biochemistry [bilirubin, creatinine, sodium, potassium, alanine-amino transferase, aspartate-amino transferase, alkaline phosphatase (AP), gamma-glutamyltransferase (γ-GT), acute phase reactants (erythrocyte sedimentation rate (ESR), C-reactive protein (CRP), fibrinogen) and urinalysis] were obtained weekly, beginning at day 1. On day 1, before the first rHIL-6 administration, blood was obtained for the measurement of baseline IL-6 concentrations (Quantikine, R&D Systems, Minneapolis, MN, USA; detection limit 0.003 ng $ml^1$). On days 1, 22 and 42 blood was sampled for qualitative and, if positive, quantitative analysis of anti-rhIL-6 antibodies. For the qualitative analysis of anti-IL-6 antibodies a 'screen ELISA' was used. In short, microtitre plates, coated with SDZ ILS 969 in coating buffer, were incubated with positive and negative antibody controls and with heat-inactivated patient serum in triplicate. After incubation alkaline phosphatase-conjugated goat anti-human IgG + IgM + IgA was added. After addition of *p*-nitrophenyl phosphate in diethanolamine optical density was read using an UVmax microtitre plate reader at 405–650 nm. An optical density of ⩽0.305 was found in 95% of normal individuals, and considered the cut-off for being antibody-negative for SDZ ILS 969. If a higher value was found, a quantitative analysis of anti-IL-6 antibodies was performed, using ELISA. Microtitre plates were coated as described above, either with coating buffer alone or with coating buffer containing SDZ ILS 969. Serum samples and controls were added in 3-fold serial dilutions (starting at 1:100) and processed as described. The specific optical density (OD) was defined as the OD of the sample in the well that was coated with SDZ ILS 969 minus the OD of the sample in the well that was coated with coating buffer only. For a claim of immunogenecity, a patient had to have a treatment-specific OD value of at least twice the pretreatment specific OD value. Values of the quantitative analysis were expressed as positive at a given titre.

Neutralising antibodies to rhIL-6 were detected using a IL-6 growth-dependent cell line (B13.29), with neutralising antiserum obtained from a goat as a positive control. A serum sample was considered as positive for neutralising antibodies if it inhibited the proliferative response to 5 units $ml^{-1}$ rhIL-6, and if the percentage inhibition was at least twice the percentage inhibition of the pretreatment serum sample at the equivalent dilution.

*Response criteria*

Only patients who completed the 6 week treatment period were included in the evaluation. A complete response was defined as the total disappearance of all detectable malignant disease for at least 4 weeks. A partial response was defined as a ⩾50% reduction of (the sum of) the product(s) of the longest diameter and the greatest perpendicular diameter of a given lesion(s) (diameter product), and no appearance of new lesions for at least 4 weeks. A minor response was defined as a reduction of ⩾25% but <50% by the same definitions. Stable disease was noted when the (sum of the) diameter product(s) decreased <50% or increased <25%. Progressive disease was defined as an increase ⩾25% of the diameter product(s) of a measurable lesion, in the sum of the products of individual lesions or in case of the appearance of new lesions. Tumour status at entry was defined by the same criteria using measurements obtained in the 3 months preceding study entry, whenever available.

*Statistics*

To assess anti-tumour activity, an Optimal Two-Stage Early Rejection Design (Simon, 1987) was used: assuming a 20% response rate would indicate treatment effectiveness, 12 patients were to be recruited in each group in the first stage. If one or more responses were observed, the trial would enter a second stage and a total (per group) of 37 patients would be recruited. All laboratory data are presented as mean ± s.e.m. Changes in any laboratory parameter during the rhIL-6 treatment were evaluated using analysis of variance, and, where appropriate, by Newman–Keul's test for multiple comparisons. Differences between groups were evaluated by Mann–Whitney's *t*-test for unmatched samples. A *P*-value <0.05 (two-tailed) was considered to represent a statistically significant difference.

### Results

*Patient characteristics*

The demographic and disease characteristics of the patients are given in Table I. A total of 49 patients was included; 12 patients with, and 37 patients without previous immunotherapy. There were no significant differences between the two groups. Of the previously treated patients, five received IL-2, four IL-2 + α-interferon (α-IFN) + lymphokine-activated killer cells, and three received α-IFN. The median interval until start of the IL-6 treatment was 8 (range 3–32) months. Two patients had a partial remission, whereas none of the others responded.

*Tumour responses*

The responses to rhIL-6 treatment are given in Table II. In the group that had had previous immunotherapy, 10 out of 12 patients were evaluable for response. There were no minor, partial or complete responses observed. Stable disease was noted in five patients. Of the five patients who had stable

**Table I** Patient characterisitcs

| | *I* | *II* | P-*value* *I* vs *II* |
|---|---|---|---|
| Number of patients | 12 | 37 | |
| Age (years) | 59 ± 2 | 61 ± 2 | NS |
| Sex (male/female) | 8/4 | 29/8 | NS |
| Time from diagnosis to treatment (months) | 27 ± 9 | 29 ± 8 | NS |
| Tumour status[a] (stable/progressive) | 5/4 | 11/15 | NS |
| Performance (0/1) | 5/7 | 21/16 | NS |
| Metastatic sites[b] | | | |
| Lung | 8 | 25 | NS |
| Bone | 1 | 7 | NS |
| Other | 7 | 15 | NS |
| Nephrectomy | 6 | 24 | NS |
| Previous radiotherapy | 5 | 10 | NS |
| Previous chemotherapy | 2 | 8 | NS |

[a] Data not available for all patients. [b] Total number may exceed number of patients owing to multiple metastatic sites in some patients. NS, not significant.

**Table II** Tumour response after 6 weeks of rhIL-6 treatment in patients with (I) and without (II) prior immunotherapy

| | *I* | *II* |
|---|---|---|
| Complete remission | – | – |
| Partial remission | – | 2 |
| Stable disease | 5 | 12 |
| Progressive disease | 5 | 16 |

disease at entry, three remained stable. Two of the four patients with progressive disease at entry had stable disease at the end of the treatment.

In the group without prior immunotherapy, two partial responses were noted. One partial response occurred in a 66 year-old male with pulmonary and liver metastases that progressed on chemotherapy with fluorodeoxyuridine, administered until 2 months before rhIL-6 treatment. A radical nephrectomy had been performed 36 months before. The patient noticed a considerable improvement in well-being while on rhIL-6 treatment. At the end of the 6 week rhIL-6 treatment course an approximately 60% tumour reduction was noted, with a further reduction during the next 10 weeks (total tumour reduction 80%). A second 6 week rhIL-6 treatment, initiated 4 months later because of recurrent disease, unfortunately failed to reinduce a remission. The other partial response was observed in a 65-year-old male who underwent a radical nephrectomy 36 months before, and who had para-aortic lymph node metastases that were progressive at study entry. After 6 weeks of rhIL-6 treatment stable disease was noted. Three months later, without any subsequent treatment, a partial remission was found on the basis of a 55% reduction in diameter product, that lasted 2 months. Of the other 35 patients in this group, 28 were evaluable for tumour response; 12 had stable disease, 16 had progressive disease. There were no minor responses. Seven of the 11 patients who had stable disease at entry, remained stable. Of the 15 patients who had progressive disease at the start of the rhIL-6 treatment, one had a partial response (see above) and four were stable throughout the treatment period.

### *Toxicity*

Side-effects are summarised in Table III. All patients were evaluable for toxicity, and since there were no differences in side-effects between patients with or without previous immunotherapy, the data of group I and II were pooled.

The main side-effects were fever (89% of the patients), favourably responding to acetaminophen in most patients; flu-like symptoms such as fatigue (22%), headache (14%) and myalgia or arthralgia (12%); nausea (37%); weight loss (37%); and hepatotoxicity, as indicated by increases in AF (63%) and γ-GT (61%). Two patients developed hypercalcaemia during rhIL-6 treatment. One patient had rapid progression of this disease. The other patient had stable disease. After institution of biphosphonate therapy his plasma calcium levels normalised. Finally, all patients had local erythema at the injection site, which subsided within 48 h. All toxicity reversed within 4 weeks after discontinuation of the rhIL-6 treatment. Nine patients had to discontinue rhIL-6 treatment prematurely, i.e. after a mean of $3\frac{1}{2}$ weeks: two patients in group I and seven in group II. The reasons for discontinuing rhIL-6 treatment were fatigue, leading to semi permanent bedrest, in two patients, accompanied by severe mental depression in one; deterioration of their general condition due to tumour progression in three patients; gross haematuria due to haemorrhage from the primary tumour in the kidney, with subsequent urosepsis, in one patient; hemiparesis due to tumour progression, for which radiation therapy had to be instituted, in one patient; fatal cerebral haemorrhage, without evidence of central nervous system metastases on CT scanning, but with a history of hypertension, in one patient; lack of ability to comply with the protocol in one patient.

**Table III** Toxicity, according to NCIC criteria, during rhIL-6 treatment. No grade 4 toxicity was observed

| *NCIC grade* | 1 | 2 | 3 |
|---|---|---|---|
| Fever | 21 | 23 | – |
| Anaemia | 19 | 8 | 1 |
| ↑ AF | 20 | 10 | 1 |
| ↑ γ-GT 15 | 10 | 5 | |
| Nausea | 14 | 2 | 2 |
| Vomiting | 2 | 2 | 1 |
| Weight loss | 17 | 1 | – |
| Fatigue | 4 | 5 | 2 |
| Headache | 7 | – | – |
| Myalgia/arthralgia | 5 | 1 | – |
| Dizziness | – | – | 1 |
| Mental depression | – | – | 1 |
| Stomatitis | 3 | – | – |
| Diarrhoea | 2 | – | – |

### *Haematology and acute phase response*

Figures 1 and 2 show the time course of the haematological effects of and the acute phase response to rhIL-6 treatment. Anaemia was a frequently noted side-effect, necessitating blood transfusions in 13 patients. Haemoglobin content progressively declined during the rhIL-6 administration by a mean of approximately 19% (from $8.5 \pm 0.2$ to $6.5 \pm 0.1$ mmol $l^{-1}$, baseline *vs* nadir $P < 0.001$). At week 10, i.e. 4 weeks after discontinuation of the rhIL-6 treatment,

**Figure 1** Changes in leucocyte (**a**) and thrombocyte (**b**) counts (in $10^9 l^{-1}$) (mean ± s.e.m.) during and after rhIL-6 treatment. $^*P < 0.05$ *vs* baseline value.

**Figure 2** Changes in fibrinogen (**a**) and CRP (**b**) concentrations (mean ± s.e.m.) during and after rhIL-6 treatment. $^*P<0.05$ *vs* baseline value.

**Figure 3** Baseline plasma IL-6 concentrations expressed as a function of tumour status at study entry (**a**) or as a function of the response to rhIL-6 treatment (**b**). Dotted line indicates detection level of the IL-6 assay at $3\,pg\,ml^{-1}$.

mean haemoglobin content in patients who did not receive a blood transfusion was still lower than the baseline value. Administration of rhIL-6 led to a significant increase in leucocyte count (from $7.3 \pm 0.3 \times 10^9\,l^{-1}$ at baseline to $8.6 \pm 0.4 \times 10^9\,l^{-1}$ at week 3, $P<0.001$) (Figure 1), without major changes in differential counts. A gradual increase of the thrombocyte count, reaching a plateau after 3 weeks (from $298 \pm 15$ to $456 \pm 17 \times 10^9\,l^{-1}$; baseline *vs* week 3, $P<0.001$) was observed (Figure 1). At week 10 the thrombocyte count was not different from baseline. RhIL-6 also induced an acute phase response, as indicated by increases in CRP (from $28 \pm 5\,mg\,l^{-1}$ at baseline to $149 \pm 7\,mg\,l^{-1}$ at week 2, $P<0.001$) and fibrinogen (from $5.58 \pm 0.26\,mg\,l^{-1}$ to $8.76 \pm 0.36\,mg\,l^{-1}$ at week 2, $P<0.001$) (Figure 2). ESR increased parallel to the increases in fibrinogen (from $47 \pm 5\,mm\,h^{-1}$ to $105 \pm 3\,mm\,h^{-1}$ at week 2, $P<0.001$). At week 10 CRP and fibrinogen concentration, but not ESR, had returned to baseline values.

### *IL-6 concentrations and immunogenecity*

Pretreatment samples for determination of IL-6 concentrations and pre- and post-treatment samples for determination of anti-IL-6 antibodies were received from 41 subjects. At baseline the IL-6 concentrations were below the detection limit in 17 subjects. Figure 3 shows the individual baseline IL-6 concentrations of the patients whose tumour status at entry was known. Furthermore, baseline IL-6 concentrations in relation to tumour response are given. From these data it can be concluded that there was no statistically significant difference in IL-6 concentrations between those who had stable or progressive disease at entry, or between those who did or did not respond to rhIL-6 treatment.

Anti-IL-6 antibodies were detected in five patients in group I and in 1 patient in group II. On quantitative analysis, the anti-IL-6 antibody titres varied from 1:100 to 1:300. Of these patients, three had stable disease after rhIL-6 treatment, and three had progressive disease. Neutralising antibodies were only detectable in the one patient with anti-IL-6 antibodies in group II. This patient had pulmonary metastases that were stable at study entry, and his disease progressed during rhIL-6 treatment.

### Discussion

This phase II trial investigated the anti-tumour effects of interleukin 6 in metastatic renal cell cancer. Two partial remissions were noted. Spontaneous tumour regression in metastatic renal cell carcinoma, especially shortly after surgical removal of the primary tumour, has been observed in less than 1% of all patients (Marston *et al.*, 1989). However, since neither of these two patients underwent a recent nephrectomy, the inhibition of tumour growth may have been the consequence of the rhIL-6 administration. Anti-tumour effects of IL-6 in experimental studies may be either direct (Chen *et al.*, 1988) or mediated by enhancing cytotoxic T-lymphocytes (Mulé *et al.*, 1992; Porgador *et al.*, 1992) or by other as yet undefined routes. A non-direct anti-tumour effect in our patients seems more likely, given the perpetuation of tumour reduction after completion of the rhIL-6 treatment. This latency is in accordance with previous findings in patients with renal cell cancer who responded to other immunotherapy (Wirth, 1993).

Toxicity, haematological and biochemical changes induced by the subcutaneous rhIL-6 treatment were largely comparable with that previously reported in humans (Van Gameren *et al.*, 1994; Weber *et al.*, 1993). In these previous studies rhIL-6 was administered during a maximum of 7 consecutive days. Prolonging the treatment period to 42 days, as in our study, revealed no tachyphylaxis for the main side-effects and laboratory changes, as was previously documented in animal studies with other cytokines (Takahashi *et al.*, 1991). Since toxicity was tolerable without requiring hospitalisation in the majority of patients, we conclude that rhIL-6 can be safely administered on an outpatient basis.

Anti-IL-6 antibodies during or after rhIL-6 treatment were detected in 15% of the patients. Half of these patients had stable disease after rhIL-6 treatment, the other half progressive disease. Neutralising antibodies were detected in only one patient. It is therefore concluded that our results were not affected by the interference of antibodies against the rhIL-6 used.

This study was initiated after observing a tumour response of pulmonary metastases in a patient with renal cell carcinoma after rhIL-6 treatment. The role of IL-6 in renal cell carcinoma, however, is complex. Paraneoplastic symptoms, such as cachexia, fever, elevated erythrocyte sedimentation rate and anaemia, commonly observed in renal cell cancer (Wirth, 1993), have been linked to the presence of elevated plasma concentrations of IL-6 in these patients (Blay *et al.*, 1992; Tsukamoto *et al.*, 1992). As a source of IL-6 the renal carcinoma itself could be appointed, since both normal (Gogusev *et al.*, 1993) and malignant renal cells (Tsukamoto *et al.*, 1992; Gogusev *et al.*, 1993) have been shown to produce and release IL-6. Moreover, *in vitro* data indicate that IL-6 may act as an autocrine growth factor in these tumours (Miki *et al.*, 1989; Gruss *et al.*, 1991; Koo *et al.*, 1992). On the other hand, IL-6 is not an independent predictor of survival as would be predicted if it were a clinically important autocrine growth factor (Stadler *et al.*, 1992). In our patient population neither endogenous baseline IL-6 concentrations nor the exogenous IL-6 administration could be related to tumour behaviour.

In two previous, preliminary reports a 5-day continuous infusion of a 15-fold higher dosage of rhIL-6 ($n = 12$), or a 14 day course of s.c. rhIL-6 administration ($n = 1$) in patients with metastatic renal cell cancer resulted in no objective responses (Ravoet *et al.*, 1994; Weiss *et al.*, 1994). We observed a partial remission in only 2 out of 49 (4%) patients. The patient characteristics of the responders in our study were not discernible from those of the non-responders. Therefore, at present rhIL-6 given as a daily s.c. injection for 6 weeks cannot be advocated as a treatment modality for metastatic renal cell carcinoma. Our data further indicate, however, that prolonged treatment with rhIL-6 can be performed safely on an outpatient basis and is associated with moderate, reversible toxicity. Finally, continuous rhIL-6 administration is accompanied by the development of non-neutralising anti-IL-6 antibodies in only a minority of patients.

### References

BLAY J-Y, NEGRIER S, COMBARET V, ATTALI S, GOILLOT E, MERROUCHE Y, MERCATELLO A, RAVOULT A AND TOURANI JM. (1992). Serum level of interleukin-6 as a prognostic factor in metastatic renal cell carcinoma. *Cancer Res.*, **52,** 3317–3322.

CHEN L, MORY Y, ZILBERSTEIN A AND REVEL M. (1988). Growth inhibition of human breast carcinoma and leukemia/lymphoma cell lines by recombinant interferon-$\beta_2$. *Proc. Natl Acad. Sci. USA*, **85,** 8037–8041.

GAULDIE J, RICHARDS C AND BAUMANN H. (1992). IL-6 and the acute phase reaction. *Res. Immunol.*, **143,** 755–759.

GOGUSEV J, AUGUSTI M, CHRÉTIEN Y AND DROZ D. (1993). Interleukin-6 and TNF$\alpha$ production in human renal cell carcinoma. *Kidney Int.*, **44,** 585–592.

GRUSS HJ, BRACH MA, MERTELSMANN RH AND HERRMANN F. (1991). Interferon-gamma interrupts autocrine growth mediated by endogenous interleukin-6 in renal cell carcinoma. *Int. J. Cancer*, **49,** 770–773.

HOUSSIAU F AND VAN SNICK J. (1992). IL-6 and the T-cell response. *Res. Immunol.*, **143,** 740–743.

ISHIBASHI T AND ASANO S. (1992). IL-6 and thrombocytopoiesis. *Res. Immunol.*, **143,** 752–754.

KISHIMOTO T AND HIRANO T. (1988). Molecular regulation of B lymphocyte response. *Ann. Rev. Immunol.*, **6,** 485–512.

KOO AS, AMSTRONG C, BOCHNER B, SHIMABUKURO T, TSO CL, DEKERNION JB AND BELLDEGRUN A. (1992). Interleukin-6 and renal cell cancer: production, regulation and growth effects. *Cancer Immunol. Immunother.*, **35,** 97–105.

LE J AND VILCEK J. (1989). Biology of disease. Interleukin-6: a multifunctional cytokine regulating immune reactions and the acute phase protein response. *Lab. Invest.*, **61,** 588–602.

MARSTON LINEHAM W, SHIPLEY WU AND LONGO DL. (1989). Cancer of the kidney and ureter. In *Cancer: Principles and Practice of Oncology*, (3rd ed.), DeVita Jr VT, Hellman S and Rosenberg SA (eds.) pp. 979–1007. J.B. Lippincott: Philadelphia.

MIKI S, IWANO M, MIKI Y, YAMAMOTO M, TANG B, YOKOKAWA K, SONODA T, HIRANO T AND KISHIMOTO T. (1989). Interleukin-6 (IL-6) functions as an in vitro autocrine growth factor in renal cell carcinomas. *FEBS Lett.*, **250,** 607–610.

MULÉ JJ, MCINTOSH JK, JABLONS DM AND ROSENBERG SA. (1990). Antitumour activity of recombinant interleukin-6 in mice. *J. Exp. Med.*, **171,** 629–636.

MULÉ JJ, CUSTER MC, TRAVIS WD AND ROSENBERG SA. (1992). Cellular mechanisms of antitumour activity of recombinant IL-6 in mice. *J. Immunol.*, **148,** 2622–2629.

PORGADOR A, TZEHOVAL E, KATZ A, VADAI E, REVEL M, FELDMAN M AND EIDENBACH L. (1992). Interleukin-6 gene transfection into Lewis lung carcinoma tumour cells suppresses the malignant phenotype and confers immunotherapeutic competence against parental metastatic cells. *Cancer Res.*, **52,** 3679–3686.

RAVOET C, DEGRÈVE J AND VANDEWOUDE K. (1994). Tumour stimulating effects of recombinant human interleukin-6. *Lancet*, **344,** 1576–1577.

REVEL M. (1992). Growth regulatory functions of IL-6 and antitumour effects. *Res. Immunol.*, **143,** 769–773.

SIMON R. (1987). How large should a phase II trial of a new drug be? *Cancer Treat. Rep.*, **71,** 1079–1085.

STADLER WM, RICHARDS JM AND VOGELZANG NJ. (1992). Serum interleukin-6 levels in metastatic renal cell carcinoma: correlation with survival but not an independent prognostic indicator. *J. Natl Cancer Inst.*, **84,** 1835–1836.

STOUTHARD JML, VAN DER POLL T, ENDERT E, BAKKER PJM, VEENHOF CHN, SAUERWEIN HP AND ROMIJN JA. (1994). Effects of acute and chronic interleukin-6 administration on thyroid hormone metabolism in humans. *J. Clin. Endocrinol. Metab.*, **79,** 1342–1346.

STOUTHARD JML, ROMIJN JA, VAN DER POLL T, ENDERT E, KLEIN S, BAKKER PJM, VEENHOF CHN AND SAUERWEIN HP. (1995). The endocrinologic and metabolic effects of interleukin-6 in humans. *Am. J. Physiol.*, **268,** E813–E819.

TAKAHASHI N, BROUCKAERT P AND FIERS W. (1991). Induction of tolerance allows separation of lethal and anti-tumor activities of tumor necrosis factor in mice. *Cancer Res.*, **51,** 2366–2372.

TSUKAMOTO T, KUMAMOTO Y, MIYAO N, MASUMORI N, TAKAHASHI A AND YANASE M. (1992). Interleukin-6 in renal cell carcinoma. *J. Urol.*, **148,** 1778–1782.

VAN GAMEREN M, WILLEMSE PHB, MULDER NH, LIMBURG PC, GROEN HS, VELLENGA E AND DE VRIES EGE. (1994). Effects of recombinant human interleukin-6 in cancer patients: a phase I-II study. *Blood*, **84,** 1434–1441.

VANTONGELEN K. (ed.) (1991). *National Cancer Institute of Canada Clinical Trials Group Expanded Common Toxicity Criteria. A Practical Guide to EORTC Studies.* pp 119–131.

WEBER JS, YANG JC, TOPALIAN SL, PARKINSON DR, SCHWARTZENRTUBER DS, ETTINGHAUSEN SE, GUNN H, MIXON A, KIM H, COLE D, LEVIN R AND ROSENBERG SA. (1993). Phase I trial of subcutaneous interleukin-6 in patients with advanced malignancies. *J. Clin. Oncol.*, **11,** 499–506.

WEISS GR, MARGOLIN KA, SZNOL M, ATKINS MB, OLEKSOWICZ L, ISAACS R AND FISHER RI. (1994). A phase II trial of a 120-hour continuous intravenous (CIV) infusion of interleukin-6 (rHuIL-6) for metastatic renal cell carcinoma (RCC). (abstract). *Proc. ASCO*, **13,** 248.

WIRTH MP. (1993). Immunotherapy for metastatic renal cell carcinoma. *Urol. Clin. N. Am.*, **20,** 283–295.

YAGODA A, PETRYLAK D AND THOMPSON. (1993). Cytotoxic chemotherapy for advanced renal cell carcinoma. *Urol. Clin. N. Am.*, **20,** 303–321.